# Silibinin inhibits the migration and invasion of human gastric cancer SGC7901 cells by downregulating MMP-2 and MMP-9 expression via the p38MAPK signaling pathway

**DOI:** 10.3892/ol.2021.12676

**Published:** 2021-03-24

**Authors:** Shuming Lu, Zhuqing Zhang, Meiru Chen, Chunyan Li, Lina Liu, Yan Li

Oncol Lett 14: 7577-7582, 2017; DOI: 10.3892/ol.2017.7080

An interested reader drew to the authors’ attention that three of the data panels for the 0 h experiments in Fig. 1 appeared to be strikingly similar, and there were also similarities comparing data panels in Fig. 2 with those featured in Fig. 4. The authors were able to consult the original data files, and realized that some inadvertent errors had been made in compiling these figures.

The corrected versions of Figs. 1 and 4 are shown on the next page (the authors have maintained that the data featured in Fig. 2 were all included as intended). Note that the errors made in the compilation of these figures did not affect the overall conclusions reported in the paper. The authors regret that these errors were not picked up upon before the paper was published, and apologize to the Editor of *Oncology Letters* and to the readership for any inconvenience caused.

## Figures and Tables

**Figure 1.**
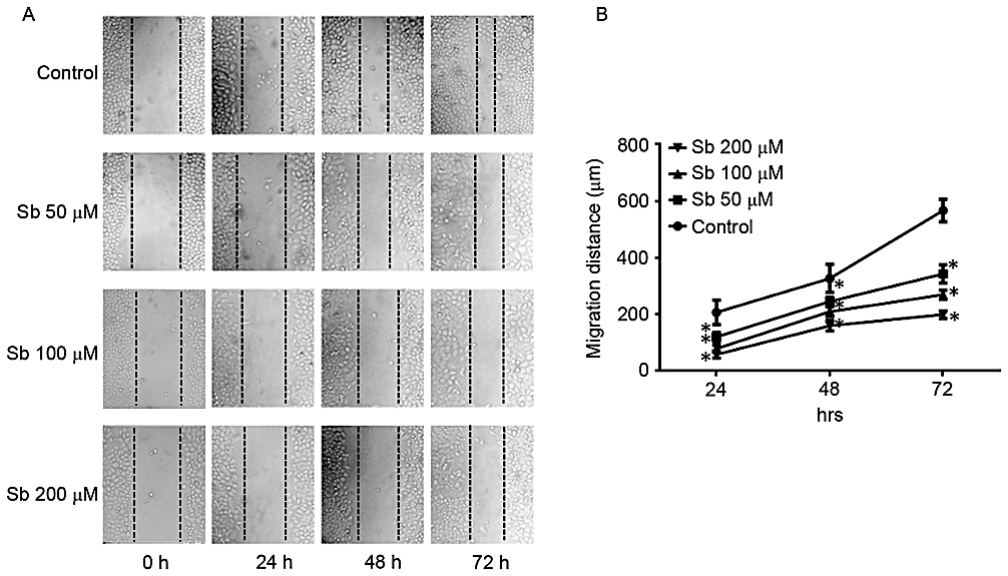
(A) Scratch assay of SGC7901 cells subsequent to treatment with 50, 100 or 200 µM Sb. Images were captured at 0, 24, 48 and 72 h at ×200 magnification under an inverted microscope. (B) Following exposure to different concentration of Sb, the migration distance of SGC7901 cells was recorded at different time points and calculated. Control cells were treated with complete medium only. *P<0.05 vs. control. Sb, silibinin.

**Figure 4.**
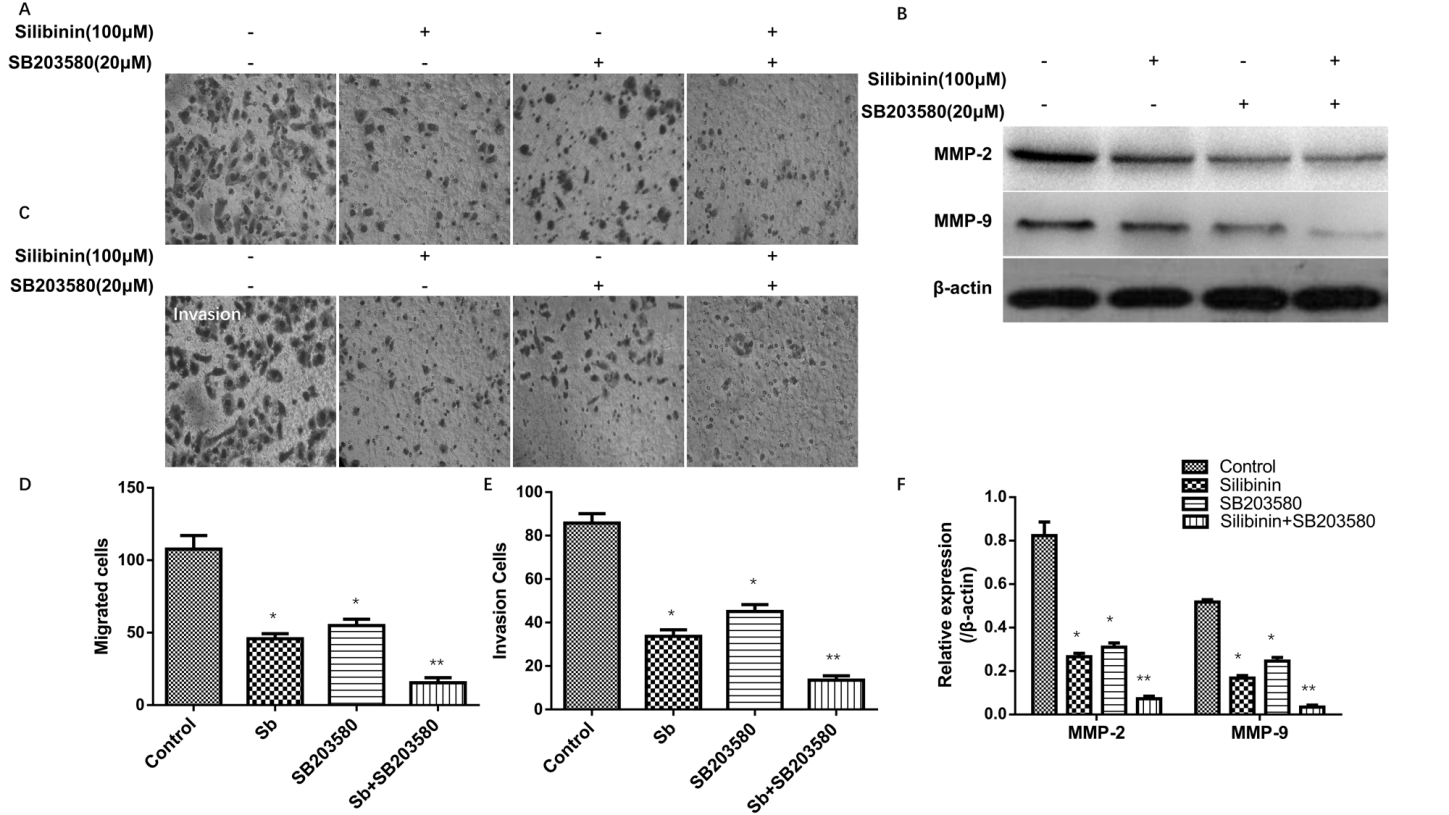
Effects of silibinin alone or in combination with SB203580 on cell migration and invasion. (A) Cell migration following treatment with silibinin alone or in combination with SB203580 (magnification, ×200). (B) The expression levels of MMP-2 and MMP-9 were (B) detected by western blot analysis. (C) Cell invasion following treatment with silibinin alone or in combination with SB203580 (magnification, ×200). Histograms present the (D) migrated cells, (E) invasive cells and (F) the quantified expression levels of MMP-2 and MMP-9. Control cells were treated with complete medium only. *P<0.05, **P<0.01 vs. control. MMP, matrix metalloproteinase; Sb, silibinin.

